# Supplementary material for: High risk of hypoxemic COVID-19 pneumonia in myasthenia gravis patients with type I IFN autoantibodies
Source: Proc Natl Acad Sci U S A. 2026 Jul 31;123(31):e2518581123. doi: 10.1073/pnas.2518581123 (PMC13439098; doi:10.1073/pnas.2518581123)
Supplement: Supplementary file 1 [file pnas.2518581123.sapp.pdf]

PNAS

High risk of hypoxemic COVID-19 pneumonia in myasthenia gravis patients with type I IFN autoantibodies

Adrian Gervais[1,2], Astrid Marchal[1,2], Alexis Maillard[1,2], Tom Le Voyer[1,2], Jérémie Rosain[1,2,3], Quentin Philipot[1,2], Lucy Bizien[1,2], Jessica Peel[4], Axel Cederholm[5], Mélanie Migaud[1,2], Sylvie Pons[6], Kahina Saker[6], Pascal Laforet[7,8], Mélodie Aubart[1,2,9], Cyril Gitiaux[9], Catherine Biggs[10], Rafael Leon Lopez[11], Sarah Souvannanorath[12], Céline Tard[13], Aleksandra Nadaj Pakleza[14], Aude-Marie Grapperon[15], Nicholas Heming[16-19], Djillali Annane[16-19], Annie Verschueren[15], Shahram Attarian[15], Kévin Bigaut[14], Karolina Hankiewicz[20], Ludivine Kouton[15], Rocio-Nur Villar-Quiles[21], Cécile Cauquil[22], Marie-Céline Fleury[14], Emilie Rocher[14], Guillaume Nicolas[7,8], Eduardo de Paula Estephan[23,24], Maria da Penha Ananias Morita[24], Edmar Zanoteli[25], Zakaria Saied[26], Amine Rachdi[26], Amouri Rim[27], Samir Belal[26], Samia Ben Sassi[26], Annemarie Hübers[28], Emmanuel Faure[29,30], Isabelle Desguerre[9], Clémence Basse[30,31], Nicolas Girard[30,31], Vivien Béziat[1,2,4], Qiang Pan-Hammarström[32], Lennart Hammarström[32], Aaron Bodansky[33], Audrey V. Parent[33], Mark S. Anderson[34], Joseph L. DeRisi[35,36], Sophie Demeret[37], Frédérique Truffault[37], Romain Fort[38], Florence Ader[39], Florent Wallet[40], Laurent Abel[1,2,4], Thierry Molina[2,41], Marie-Alexandra Alyanakian[42,&], Rozen Le Panse[37,&], Guilhem Solé[43,44,&], Aurélie Cobat[1,2,4,*], Nils Landegren[5*], Jean-Laurent Casanova[1,2,4,45,46*@], Anne Puel[1,2,4,*], Paul Bastard[1,2,4,46*,@], and Emmanuelle Jouanguy[1,2,4*]

&,*: Equal contributions

@: Correspondence: Paul Bastard (paul.bastard@institutimagine.org), Jean-Laurent Casanova (casanova@rockefeller.edu)

Supplementary methods

Patient recruitment and ethics approval

The details of the IRB/oversight body that provided approval or exemption for the research described are as follows: written informed consent for participation in this study was obtained from all participants and/or their legal guardians in accordance with local regulations, with approval from the institutional review board (IRB). For samples provided by the team (Sorbonne University/INSERM - UMRS974), investigations were approved by the local ethics committee (the relevant authorization numbers are ID RCB: 2006-A00164-47 ("Développement de modèles pour améliorer la prise en charge de la Myasthénie : de la connaissance fondamentale à l'application clinique (projet MYASTAID)", which ethics approval was granted by the French Comité Consultatif de Protection des Personnes CPP IDF VII (Kremlin Bicêtre); and 2010-A00250-39 ("Construction d'une collection de tissus pour le projet FIGHT-MG", which ethics approval was granted by the French Comité Consultatif de Protection des Personnes CPP IDF VI (Pitié Salpêtrière)). All the experiments and analyses were performed in accordance with the relevant guidelines and regulations. For the rest of the samples, ethics approval was granted by the French Comité Consultatif de Protection des Personnes - CCP - Ile-de-France VII, Kremlin Bicêtre, under protocols 2006-A00164-47 and 2010-A00250-39. Approval for the analysis of patient samples was also granted by the Institutional Review Boards at The Rockefeller University (New York, USA) and INSERM (France).

Protein array

Protein arrays (HuProt™ from CDI laboratories) were incubated in 5 mL blocking buffer, consisting of 2% bovine serum albumin and 0.05% Tween 20 in phosphate-buffered saline (PBS), for 90 min. The arrays were then incubated overnight in 5 mL blocking buffer per array with either blood donor serum or patient serum diluted 1:2000. Each array was then washed

five times, for five minutes each, with 5 mL PBST (PBS + 0.05 % Tween 20). Alexa Fluor 647 goat anti-human IgG (Thermo Fisher Scientific Cat# A-21445, RRID:AB_2535862, LOT# 2286304) and Dylight® 550 goat anti-GST (Columbia Biosciences Corporation Cat# D9-1310, LOT# DY550011-18-003,) antibodies were diluted in blocking buffer (1:2000 and 1:10 000, respectively) and each array was incubated in 5 mL of the resulting mixture for 90 min. The arrays were washed five times, as described above. Incubations and washes were performed on an orbital shaker, with tubes wrapped in aluminum foil to block out light following the addition of fluorescent antibodies. Finally, each array was immersed three times in deionized water and centrifuged for approximately 30 seconds for drying. The arrays were scanned later the same day with an Innoscan 1100AL Fluorescence scanner (Innopsys) under the control of Mapix software and the resulting images were analyzed with GenePix Pro 5.1.0.19 and HuProt_v4.0.gal. Protein names are consistent with the aforementioned file in some cases, whereas proteins are named in accordance with HuProt_v4.0_Standard_Gal_Updated_04302019.gal in others. Data were normalized to compensate for the variation of signal intensity between experiments. All patient samples were screened in the same experiment, together with samples from two healthy controls. Data from additional healthy controls were included in separate protein array experiments. Signal intensities were extracted from the scanned image with GenePix Pro 5.1.0.19, with subtraction of the local background as follows:

$$Signal_{Protein} =$$

$$\mathrm{median}(Spot\ Pixel\ Intensity_{635}) - (Background\ Pixel\ Intensity_{635})$$

Each protein was printed in duplicate spots. The resulting signal for one sample is defined as follows:

$$Signal_{Sample} = \max \begin{cases} Signal_{Protein}\ duplicate\ 1 \\ Signal_{Protein}\ duplicate\ 2 \end{cases}$$

We screened for spurious results, by screening duplicates for large differences:

$$Maximum\ duplicate\ discordance\ _{Protein} =$$

$$\max \begin{cases} Signal_{Sample\ 1}\ duplicate\ 1 - \ Signal_{Sample\ 1}\ duplicate\ 2 \\ Signal_{Sample\ 2}\ duplicate\ 1 - \ Signal_{Sample\ 2}\ duplicate\ 2 \\ \ldots \\ Signal_{Sample\ n}\ duplicate\ 1 - \ Signal_{Sample\ n}\ duplicate\ 2 \end{cases}$$

Mean signal intensity was calculated for case and control samples separately:

$$\mu_{SignalCases} = \sum_{i=1}^{n_{Cases}} Signal_{Sample}\ in\ cases$$
